# Monitoring the effect of different microwave extraction parameters on the recovery of polyphenols from shiitake mushrooms: Comparison with hot-water and organic-solvent extractions

**DOI:** 10.1016/j.btre.2020.e00504

**Published:** 2020-07-08

**Authors:** Wen Xiaokang, James G. Lyng, Nigel P. Brunton, Lydia Cody, Jean-Christophe Jacquier, Sabine M. Harrison, Konstantinos Papoutsis

**Affiliations:** UCD School of Agriculture and Food Science, University College Dublin, Belfield, Dublin 4, Ireland

**Keywords:** Fungi, Sustainable extraction, Particle size, Antioxidants, Scanning electron microscopy

## Abstract

The current study aimed to investigate the effect of different microwave-assisted extraction (MAE) parameters (i.e., particle size of the sample, solid-to-liquid ratio, microwave power, and extraction time) on the total phenolic content (TPC), antioxidant capacity (DPPH and CUPRAC), chlorogenic acid and caffeic acid contents of shiitake mushrooms. All the independent variables affected TPC and antioxidant capacity values. Only the sample particle size had no significant effect on phenolic acid contents. The highest TPC, DPPH, and CUPRAC values were obtained when a particle size of 1.75 mm, solid-to-liquid ratio of 1/40, microwave power of 600 W, and extraction time of 15 min were used. The extracts obtained by MAE were compared with those obtained after hot-water extraction (HWE) and organic-solvent extraction (OSE). Scanning electron microscopy (SEM) confirmed that MAE resulted in cell wall disruption which might be due to an increase in the pressure of the inner part of the cells.

## Highlights

• MAE facilitates the extraction of phenols from mushrooms in short processing times.
• Three extraction methods were compared.
• Chlorogenic and caffeic acids were identified in the MAE extracts.
• SEM showed that all the extraction methods led to cell damage to varying extents.

## Graphical abstract

**Figure:**
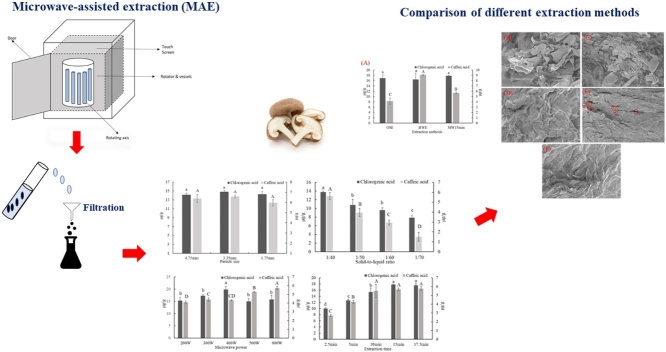

## 1 Introduction

Mushroom global consumption has increased from 1 to 4.7 kg of cultivated edible mushrooms per capita from 1997 to 2013 [1]. According to the Food and Agriculture Organization of the United Nations (FAO), global mushroom production in 2016 reached nearly 11 million tons [2]. High amounts of mushroom by-products are generated during mushroom production which may account for up to 20 % and are mainly composed of mushrooms that their caps and/or stalks are misshaped and hence do not meet the specifications set by retailers [[3], [4], [5]]. Several studies have noted that mushrooms exhibit beneficial properties for human health, which have been attributed to their content in secondary metabolites, such as polyphenols, sterols, and polysaccharides [6,7]. Therefore, mushroom by-products could be used as a substrate for the recovery of compounds that could be valorized by pharmaceutical industries adding value to the horticultural sector.

Polyphenols are metabolites containing one or more aromatic rings with one or more hydroxyl groups and are known for their antioxidant capacity since they reduce the action of reactive oxygen species (ROS) [[8], [9], [10]]. Polyphenols in mushrooms belong to phenolic acids and flavonoids and their content varies among the different species [[11], [12], [13], [14], [15]]. Caffeic and chlorogenic acids belong to hydroxycinnamic acids and have attracted scientific interest due to their health beneficial properties [15,16]. Chlorogenic acid is the ester of caffeic acid and quinic acid [17]. Both phenolic acids have been previously reported in shiitake and other mushroom species [15,18].

Extraction is the first step for the recovery of bioactive compounds from samples. Different extraction methods can be employed namely conventional and non-conventional. Conventional extraction methods have the disadvantages of high solvent requirements, thus, a large amount of solvent waste generation, as well as high energy consumption. These disadvantages can be overcome by the use of non-conventional extraction methods, including microwave-assisted extraction (MAE), ultrasound-assisted extraction (UAE), enzyme-assisted extraction, subcritical and supercritical fluid extraction [5,7,19,20].

MAE uses microwaves which are irradiation of the electromagnetic spectrum ranging in frequency from 300 MHz to 300 GHz [21]. The process of MAE can be summarized as follows: deprivation of solutes from the active sites in sample matrix under increasing temperature and pressure conditions, diffusion of extraction solvent into sample matrix, and dissolution of solutes in the solvent [22]. During MAE the recovery of bioactive compounds occurs due to material direct heating caused by dipole polarization and ionic conduction and starts from the interior part of the matrix [23]. Specifically, microwave energy is absorbed by polar molecules and the internal heating causes liquid vaporization and pressure built up within the cells [21,23].

To the best of our knowledge, to date, two studies have investigated the effects of different MAE parameters on polyphenol recovery from mushrooms. Specifically, Maeng et al. [24] investigated the impact of MAE conditions (extraction time, ethanol concentration, and microwave power) on the recovery of TPC and antioxidants from *Coriolus versicolor* using response surface methodology (RSM). Zhang et al. [14] investigated the effects of MAE conditions, including ethanol concentration, solid-to-liquid ratio, irradiation time, extraction temperature, irradiation power, and extraction cycle on the recovery of six phenolic compounds from *Agaricus blazei*. However, there is no study investigating the impact of different MAE parameters on polyphenols (total and individual contents) and the antioxidant capacity of shiitake mushrooms.

The current study aimed to explore the effects of different MAE parameters (i.e., particle size of the sample, soli-to-liquid ratio, microwave power, and extraction time) on the polyphenols (total and individual) and antioxidant capacity from shiitake mushrooms by monitoring the influence of one parameter at a time. Subsequently, the extracts obtained from the MAE compared with those obtained following hot-water extraction (HWE) [25] and organic-solvent extraction (OSE) [26]. Scanning electron microscopy (SEM) was employed for observing potential changes in mushroom residues after extraction.

## 2 Materials and methods

### 2.1 Chemicals

Copper chloride, 2,2-diphenyl-1-picrylhydrazyl (DPPH), 6-hydroxy-2,5,7,8-tetramethylchroman-2-carboxylic acid (trolox), neocuproine, ammonium acetate, caffeic acid, chlorogenic acid, gallic acid, rutin, quercetin, apigenin, sodium carbonate and Folin-Ciocalteu’s reagent were obtained from Sigma-Aldrich Co. (Arklow, Ireland). Ethanol, methanol, and acetic acid were of HPLC grade and were purchased from Fisher Scientific (Dublin, Ireland).

### 2.2 Materials

*Lentinula edodes* (shiitake) mushrooms were kindly provided by Commercial Mushroom Producers Ltd (C.M.P., Co. Monaghan, Ireland). Initially, mushrooms were stored at 4 °C for one day, and subsequently, all the samples were cooled under liquid nitrogen and then dried by freeze-drying for 48 h. The dried samples were ground using a commercial blender and sieved using mesh sieves of three different sizes (i.e., 1.75, 3.35, and 4.75 mm) (Endecotts Ltd., London, England).

### 2.3 Experimental design

**Fig. 1:**
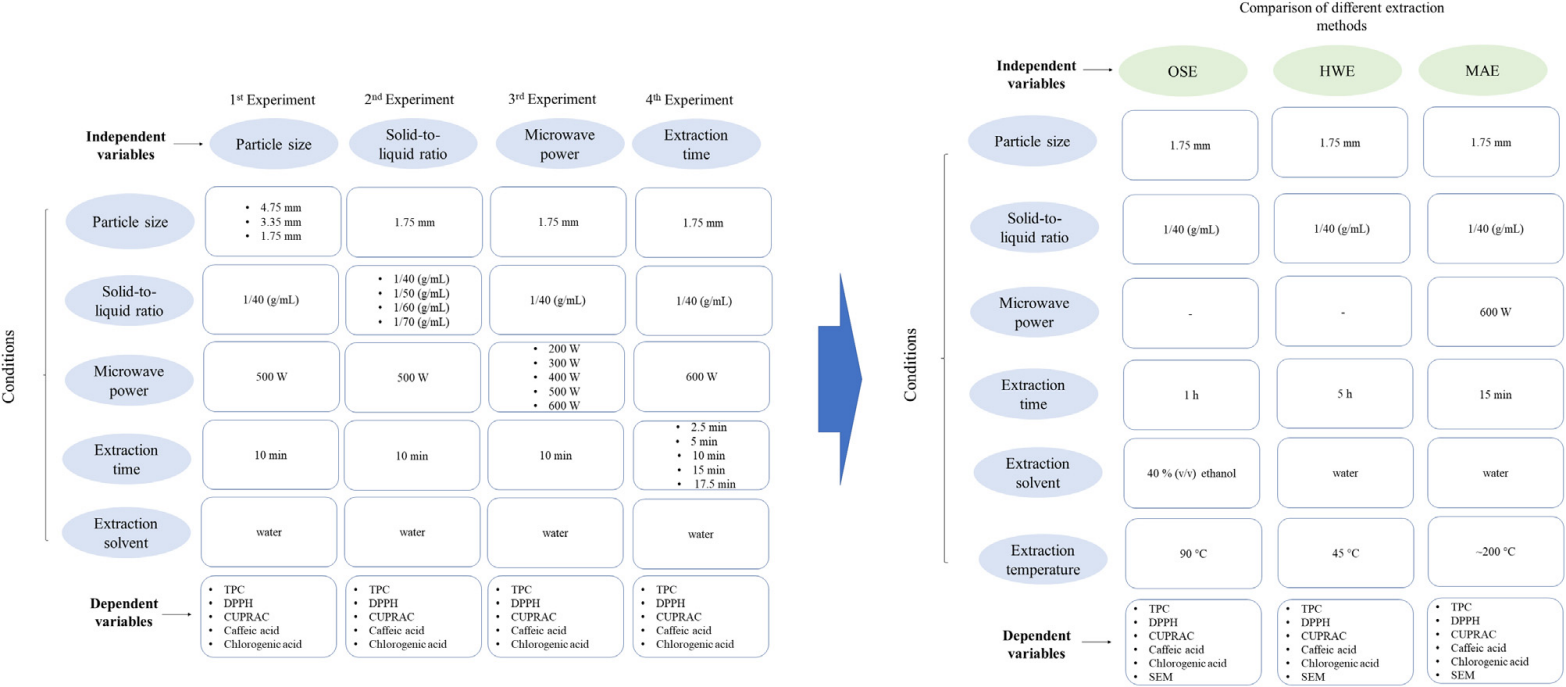
Experimental design of the study.

RSM is a multivariate statistical technique that is widely used for the optimization of an extraction process and the investigation of interaction and quadratic effects between different independent variables. However, before the application of RSM, it is necessary to select the independent variables which have significant effects on the system by conducting one factor at a time experiment [27]. In the current study, the one factor at a time strategy was selected to examine the effects of four parameters (particle size of the sample, solid-to-liquid ratio, microwave power, extraction time) during MAE. When one parameter was examined, the others were kept constant (Fig. 1). These results can be an input for future RSM studies that aim to optimize the recovery of polyphenols and the antioxidant capacity of mushrooms. Subsequently, the MAE method was compared with a HWE [25] and an OSE [26].

### 2.4 Microwave extraction of bioactive compounds from mushrooms

For the microwave extraction a MARS 6™ microwave system (CEM, Buckingham, UK) equipped with an IR sensor from below for temperature measurement, was used. After the extraction, the samples were placed in an ice to cool down and then filtered through Whatman paper number 1. The extracts were stored at 4 ℃ until analysis.

### 2.5 Hot-water extraction (HWE) and organic-solvent extraction (OSE)

The HWE was conducted according to Yim et al. [25] with few modifications. HWE was carried out for 5 h, at an extraction temperature of 45 °C using a water bath with a solid-to-liquid ratio of 1/40 (g/mL). The OSE was performed according to Zhang et al. [26] with few modifications. Specifically, 1 g of sample was mixed with 40 mL of 40 % (v/v) ethanol and incubated at 90 °C for 1 h.

### 2.6 Chemical analysis

#### 2.6.1 Total phenolic content (TPC)

The TPC was determined as described by Papoutsis et al. [28] with some modifications. Briefly, 0.5 mL of sample extraction was mixed with 2.5 mL of 1/10 (v/v) Folin-Ciocalteu’s reagent. After 5 min incubation at ambient temperature (∼22 °C), 2 mL of 7.5 % (w/v) sodium carbonate solution was added and then incubated at room temperature for 60 min. The absorbance of the sample was measured at 765 nm using a V-650 spectrophotometer (Jasco Inc. Easton, MD, USA). The results were expressed as mg of gallic acid equivalents per g (mg GAE/g). A standard calibration curve with a range of 10–100 μg/mL was used (R^2^ = 0.999).

#### 2.6.2 Antioxidant capacity

Two assays were employed for determining the antioxidant capacity of the extracts namely 2,2-diphenyl-1-picrylhydrazyl (DPPH) and cupric ion reducing antioxidant capacity (CUPRAC). Specifically, DPPH assay was determined according to Papoutsis et al. [28]. Briefly, 2.85 mL of DPPH working solution was mixed with 150 μL of sample. The mixture was incubated for 30 min in the dark at room temperature before measuring the absorbance at 515 nm. The results were expressed as mg trolox equivalents per g (mg TE/g). A standard calibration curve with a range of 100–1000 μM was used (R^2^ = 0.998). CUPRAC was determined according to Apak et al. [29]. Briefly, 1 mL of 10 mM copper chloride was mixed with 1 mL of 7.5 mM of neocuproine solution and 1 mL NH_4_Ac (pH 7.0). Subsequently, 1.1 mL of sample was added to the mixture. After incubating for 90 min at ambient temperature, the absorbance of the sample was measured at 450 nm. The results were expressed as mg trolox equivalents per g (mg TE/g). A standard calibration curve with a range of 25–200 μM was used (R^2^ = 0.995).

#### 2.6.3 High-performance liquid chromatography (HPLC)

An Agilent 1200 HPLC system (Palo Alto, CA, USA) fitted with a diode array detector (DAD) was used for the identification and quantification of individual phenolic compounds. The analysis was conducted as described by Ndungutse et al. [30]. The extracts were filtered through a 0.45 μm nylon filter before analysis. 50 μL of the filtered extract was injected onto an ACE Excel 5 C_18_-PFP column (150 × 4.6 mm) (Phenomenex, Macclesfield, UK) equipped with a guard column. The column temperature was fixed at 25 ℃. Two mobile phases were used: 0.1 % (v/v) acetic acid in water (Mobile phase A) and 100 % (v/v) methanol (Mobile phase B) with the following gradient elution protocol: 0 min, 98 % mobile phase A; 40 min, 0 % mobile phase A; 43 min, 98 % mobile phase A; 53 min, 98 % mobile phase A. The concentration of individual compounds was determined and quantified based on the external standard method using peak areas at 280 nm.

### 2.7 Scanning electron microscopy (SEM)

SEM was conducted for observing the morphology of shiitake residues after applying the three different extraction methods (MAE, HWE, and OSE) using a Hitachi regulus 8230 (Japan) equipped with an Oxford EDS170 detector (UK) according to Papoutsis et al. [28]. After extraction, shiitake mushroom residues were dried at 60 °C until constant weight. To investigate any potential effect of drying temperature on mushrooms, two controls were used: freeze-dried shiitake mushrooms (control 1) and freeze-dried shiitake mushrooms put at 60 °C (control 2). Samples were iridium coated before the images were taken.

### 2.8 Statistical analysis

The results are expressed as mean ± standard deviation. All the treatments were conducted in triplicate and compared by one-way ANOVA and Duncan’s post hoc multiple comparison test at a significance level of *p* < 0.05. The Pearson’s correlation (*r*) and *P*-value were used to determine the correlation coeﬃcients among TPC and antioxidant capacity assays.

## 3 Results and discussion

### 3.1 Effect of sample particle size on total phenolic content (TPC) and antioxidant capacity

The effect of the different parameters on the TPC and antioxidant capacity measured by DPPH and CUPRAC are seen in Table 1. The particle size of the sample had a significant impact on TPC and antioxidant capacity during MAE (*p* < 0.05). Specifically, as the particle size decreased from 4.75 to 1.75 mm, the TPC and CUPRAC values increased (from 4.90±0.13 to 6.35±0.12 mg GAE/g and from 6.67±0.15 to 8.52±0.21 mg TE/g, respectively). The lowest DPPH values determined in the extracts obtained from the particle size of 4.75 mm, while there was no significant difference between the particle sizes of 3.35 and 1.75 mm. These results are in contrast with those reported by Veljovic et al. [31] who showed that particle size had no significant linear effect on the TPC values of *Ganoderma lucidum* ethanolic extracts. However, in the same study, it was reported that particle size of the sample had a significant impact on the antioxidant capacity of the mushroom extracts measured by DPPH, ferric ion reducing antioxidant power (FRAP), and trolox equivalent antioxidant capacity (TEAC) assays [31]. Similarly to our results, Papoutsis et al. [32] noted that particle size of the sample had a significant impact on the TPC values and antioxidant capacity (measured by DPPH and CUPRAC) of aqueous extracts from citrus by-products. A decrease in particle size by milling and grinding facilitates greater destruction of cell walls, resulting in the reduction of inner mass transfer limitations, as well as easier access of the solvent to plant metabolites located in the cell walls or in the cytoplasm [21]. Moreover, as the particle size decreases the surface area in contact with the solvent increases resulting in greater mass transfer from the solid material to liquid, due to the greater penetration of the solvent into the sample tissue [32]. As a result of these observations, a particle size of 1.75 mm was selected for the following experiments. In future optimization experiments, it is recommended that the particle size of the sample should be included in an RSM design since it significantly affects the recovery of TPC. Given that the results of this study showed that higher TPC yields are obtained as the particle size of the sample decreases, the effect of smaller particle sizes (i.e., 0.50 mm, 1.00 mm) is encouraged for investigation.

**Table 1:** Effect of different parameters on the MAE of total phenolic content (TPC) and antioxidant capacity measured by DPPH and CUPRAC. The results are expressed as mean ± standard deviation (n = 3).

X1 (mm)	TPC (mg GAE/g)	DPPH (mg TE/g)	CUPRAC (mg TE/g)	X2 (g/mL)	TPC (mg GAE/g)	DPPH (mg TE/g)	CUPRAC (mg TE/g)	X3 (W)	TPC (mg GAE/g)	DPPH (mg TE/g)	CUPRAC (mg TE/g)	X4 (min)	TPC (mg GAE/g)	DPPH (mg TE/g)	CUPRAC (mg TE/g)
4.75	4.90 ± 0.13^c^*	4.54 ± 0.15^b^	6.67 ± 0.15^c^	1/40	6.07 ± 0.14^a^	4.68 ± 0.04^a^	6.52 ± 0.29^a^	200	5.73 ± 0.13^d^	5.11 ± 0.26^b^	7.80 ± 0.07^d^	2.5	4.83 ± 0.19^d^	4.36 ± 0.25^c^	6.50 ± 0.08^d^
3.35	5.71 ± 0.08^b^	5.22 ± 0.12^a^	8.00 ± 0.23^b^	1/50	5.36 ± 0.34^b^	4.65 ± 0.48^a^	5.82 ± 0.18^b^	300	5.08 ± 0.45^d^	4.15 ± 0.05^c^	7.09 ± 0.21^d^	5	6.75 ± 1.19^c^	5.13 ± 0.06^b^	9.41 ± 0.42^c^
1.75	6.35 ± 0.12^a^	5.22 ± 0.00^a^	8.52 ± 0.21^a^	1/60	5.10 ± 0.09^b^	4.77 ± 0.47^a^	5.75 ± 0.30^b^	400	6.72 ± 0.27^c^	5.54 ± 0.55^b^	10.04 ± 0.51^c^	10	8.95 ± 0.20^b^	5.76 ± 0.59^b^	12.33 ± 0.27^b^
				1/70	5.20 ± 0.41^b^	4.30 ± 0.51^a^	5.66 ± 0.15^b^	500	7.89 ± 0.69^b^	6.56 ± 0.32^a^	11.89 ± 1.27^b^	15	11.23 ± 1.05^a^	7.19 ± 0.24^a^	15.43 ± 0.56^a^
								600	10.87 ± 0.29^a^	5.27 ± 0.13^b^	15.82 ± 0.27^a^	17.5	9.66 ± 0.38^b^	5.64 ± 0.09^b^	12.90 ± 0.52^b^

X1: Particle size; X2: Solid-to-liquid ratio; X3: Power; X4: Extraction time.

* Values at the same column followed by different superscript letter within the same column are significantly different at *p* < 0.05 according to ANOVA and Duncan’s test.

### 3.2 Effect of solid-to-liquid ratio on TPC and antioxidant capacity

**Fig. 2:**
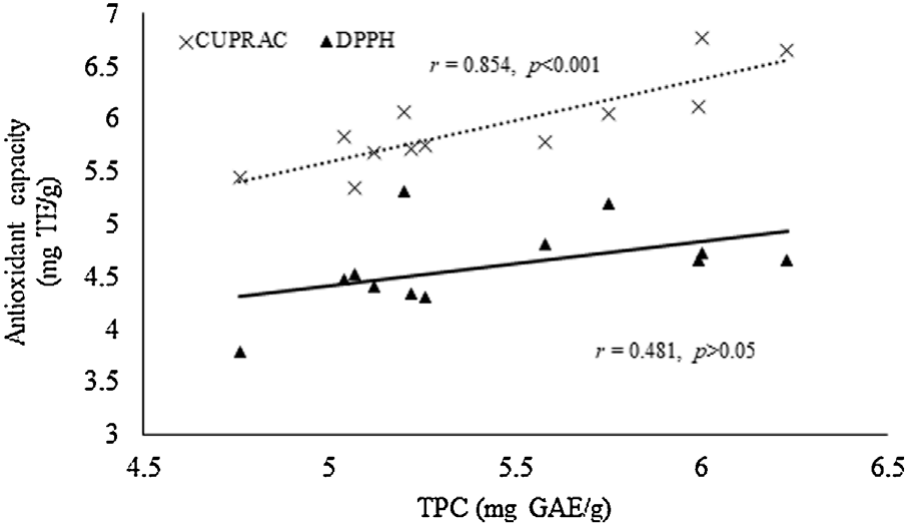
Pearson’s correlation (r) between antioxidant capacity determined by CUPRAC and DPPH and total phenolic content (TPC).

The solid-to-liquid ratio significantly affected the TPC values and antioxidant capacity of shiitake mushroom extracts during MAE (*p* < 0.05) (Table 1). As the solid-to-liquid ratio increased from 1/40 to 1/50 the TPC value and CUPRAC values significantly decreased, while there was no effect on the antioxidant capacity measured by DPPH (*p* > 0.05). This can also be justified by the high correlation found between TPC and CUPRAC, while there was no correlation between TPC and DPPH (Fig. 2). These results could be attributed to the higher concentration of polyphenols and antioxidants in the extract where a lower amount of solvent was used. Similar to our results, previous studies have noted that solid-to-liquid ratio had a significant impact on the extraction of bioactive compounds from *A. blazei* [14] and grape pomace [33]. Pinelo et al. [33] noted that higher total polyphenol values from grape pomace were obtained when a lower solvent-to-solid ratios were employed which is in accordance with the results reported in the current study. In contrast, Zhang et al. [14] mentioned that during MAE the yields of six phenolic compounds increased as the solid-to-liquid ratios decreased. However, the authors mentioned no difference in the yields obtained with the 1/30 to 1/50 ratios [14]. Based on the results obtained in this study, the solid-to-liquid ratio is a parameter that could be excluded from an RSM design in future studies aiming to optimize the recovery of TPC and antioxidants from shiitake mushrooms. From a sustainability point of view, lower solid-to-liquid ratios not only result in higher TPC and antioxidant capacity values but also less solvent consumption and solvent waste generation. The solid-to-liquid ratio of 1/40 was selected and used for the following experiments since it resulted in the highest TPC and antioxidant capacity values.

### 3.3 Effect of microwave power on TPC and antioxidant capacity

**Fig. 3:**
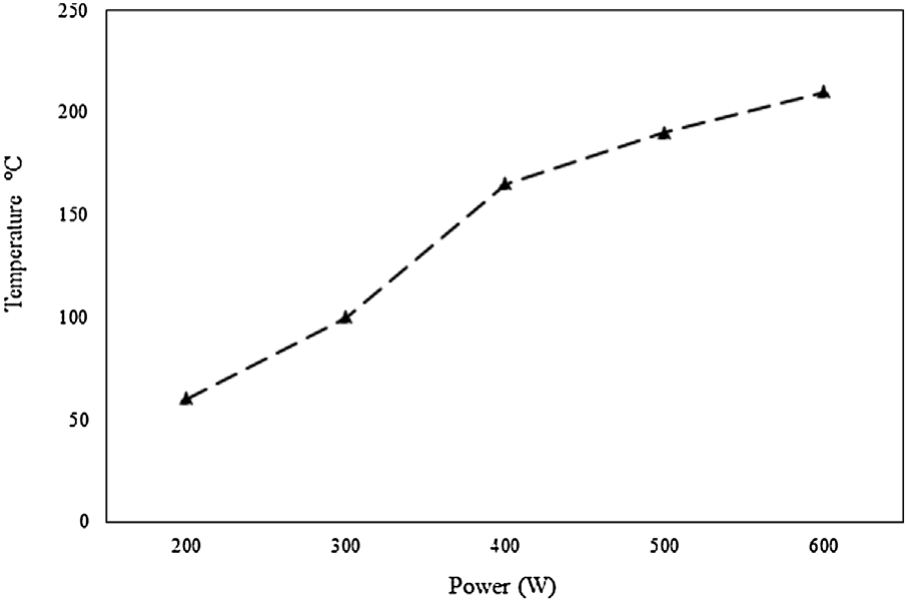
Correlation between microwave power (W) and temperature (°C).

The microwave power had a significant impact on TPC values and antioxidant capacity (Table 1) (*p* < 0.05). As the microwave power increased from 300 to 600 W the TPC values and antioxidant capacity measured by CUPRAC significantly increased (from 5.08±0.45 to 10.87±0.29 mg GAE/g, respectively) and (from 7.09±0.21 to 15.82±0.27 mg TE/g, respectively), respectively. However, DPPH values decreased when the microwave power increased from 500 to 600 W (from 6.56±0.32 to 5.27±0.13 mg TE/g, respectively). In MAE, microwaves heat the inner part of the samples leading to pressure increase inside the cells, resulting in cell wall disruption, hence, the release of bioactive compounds [23]. Fig. 3 shows the correlation between the microwave power applied and temperature increase. As the microwave power increased from 200 to 600 W, the temperature increased from 60 to 210 °C, which explains the higher TPC values obtained at 600 W. The increasing temperature not only promotes the separation of bioactive compounds from sample matrix but also results in the higher solubility of solutes due to the lower surface tension and solvent viscosity [34]. However, high extraction temperatures may result in the degradation of some thermolabile bioactive compounds with antiradical activity. These results are in accordance with Zhang et al. [14] who found that during MAE, high microwave power (500 W) enhanced the yields of phenolic compounds of *A. blazei*, while at higher microwave power (700 W) the yields declined. Similarly, Maeng et al. [24] also reported that the microwave power significantly affected the TPC values and antioxidant capacity of *Coriolus versicolor* mushroom extracts. The microwave power is a parameter that should be included in an RSM design in future studies aiming to optimize the recovery of TPC and antioxidants from shiitake mushrooms. Microwave power greater than 600 W are encouraged to be investigated since an upward trend was observed in the case of TPC and CUPRAC values. Indeed, higher microwave power may result in the degradation of some thermolabile compounds with antioxidant properties. However, this negative effect might be eliminated by the application of shorter MAE times. An RSM design including the microwave power and extraction time will allow the investigation of interaction effects between those two parameters. The power of 600 W was selected and used for the following experiments.

### 3.4 Effect of extraction time on TPC and antioxidant capacity

The extraction time during MAE is an important parameter affecting the recovery of bioactive compounds from the samples [23]. Extraction time had a significant effect on TPC values and antioxidant capacity (*p* < 0.05) (Table 1). Specifically, as the extraction time increased from 2.5 to 15 min the TPC values and antioxidant capacity increased, while an extraction time of 17.5 min resulted in the decrease of the dependent variables. This result could be attributed to the degradation of some phenolic compounds and antioxidants due to high temperatures (Fig. 3). This is in accordance with Maeng et al. [24] who mentioned that the extraction time during MAE significantly affected the TPC values and antioxidant capacity of *Coriolus versicolor* mushroom extracts. Similar results were reported by Zhang et al. [14] who noted that during MAE the yields of phenolic compounds of *A. blazei* significantly increased from 1 to 5 min and then slightly decreased. As previously mentioned, in MAE, microwaves heat the inner part of the samples leading to pressure increase inside the cells, resulting in cell wall disruption, hence, the release of bioactive compounds [23]. The more time the samples are exposed to microwave energy, the greater disruption of cell walls occurs, resulting in mass transfer from the interior of the sample to the solvent. However, prolong extraction times might result in the degradation of some bioactive compounds due to their exposure to high temperatures. In the current study, the highest TPC values and antioxidant capacity were obtained at a microwave power of 600 W for 15 min extraction time. Future studies are encouraged to employ RSM to investigate interaction effects between extraction time and microwave power. As discussed in a previous section, the application of microwave power greater than 600 W may result in shorter extraction times.

### 3.5 Effect of MAE on the recovery of caffeic and chlorogenic acids

**Fig. 4:**
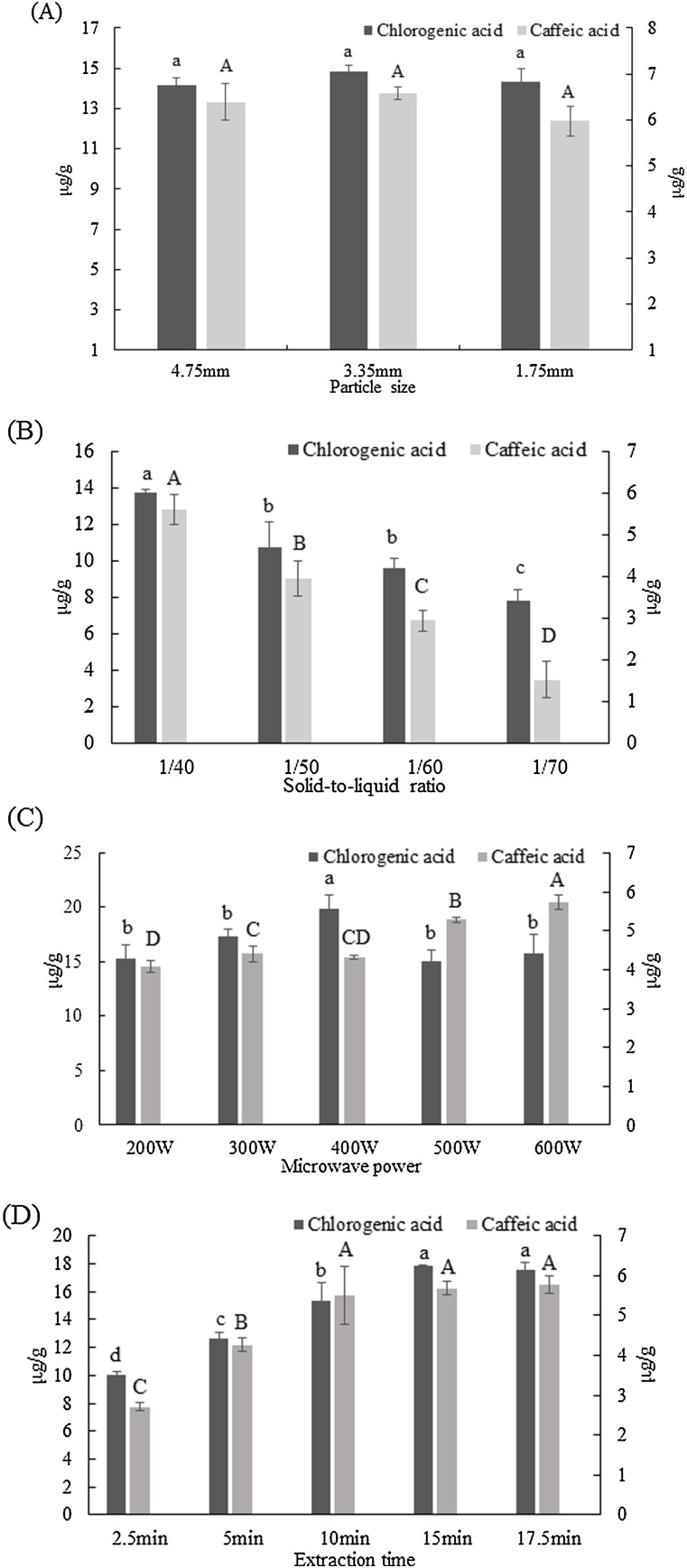
Effect of particle size of the sample (A), solid-to-liquid ratio (B), microwave power (C), and extraction time (D) on chlorogenic acid (primary left vertical axis) and caffeic acid (secondary right vertical axis). Results are expressed as mean standard deviation. Bars of the same color followed by different letters are significantly different at *p* < 0.05 according to ANOVA and Duncan’s test.

In the current study among the six phenolic compounds that were tested (i.e., caffeic acid, chlorogenic acid, gallic acid, rutin, quercetin, and apigenin), caffeic acid and chlorogenic acid were consistently identified in all the samples. Therefore, the results related to those phenolic acids are presented. The presence of caffeic and chlorogenic acids in shiitake and other mushroom species has been previously reported [15,18]. Chlorogenic and caffeic acid contents ranged between 7.83 and 19.87 μg/g and 1.51 and 5.78 μg/g, respectively. These values are close to those that have been previously reported in different mushroom species [10]. The effect of particle size was initially investigated (Fig. 4A). Even though particle size had a significant effect on the TPC values, it did not affect the recovery of phenolic acids. Caffeic and chlorogenic acids account for a fraction of the TPC [15]. It could be speculated that phenolic acids of lower molecular weights than caffeic acid and chlorogenic acid might be migrated faster from the in part of the vacuole (where they are usually found in a bound form) into the solvent. Papoutsis et al. [32] showed that during ultrasound-assisted extraction, particle size did not affect the recovery of chlorogenic acid from citrus by-products. Similar to TPC and antioxidant capacity, the solid-to-solvent ratio had a significant effect on the MAE of both phenolic acids (Fig. 4B). Higher yields of phenolic acids were obtained when a lower amount of solvent was used. These results are in contrast to those reported by Zhang et al. [14] who noted that during MAE, as a solid-to-liquid ratio decreased the yields of six phenolic compounds from *A. blazei* increased. Subsequently, the effect of microwave power was investigated (Fig. 4C). Among the different microwave power investigated (200−600 W) the highest chlorogenic acid yields were obtained at 400 W (19.87 ± 1.26 μg/g) while the highest caffeic acid yields at 600 W (5.73 ± 0.20 μg/g). As shown in Fig. 3, an increase in microwave power resulted in a temperature increase in the samples, which may explain the reduced extraction yields of chlorogenic acid obtained when 500 and 600 W were applied. Chlorogenic acid is a thermally unstable compound, and at high temperatures is decomposed to quinic acid and caffeic acid [35]. This could be the explanation of obtaining greater caffeic acid yields at 500 and 600 W microwave power in parallel to the decline in yields observed for chlorogenic acid. Extraction time significantly affected the recovery of both phenolic acids. As the extraction time increased from 2.5 to 15 min, the chlorogenic acid yields increased (from 10.01 ± 0.24 to 17.81 ± 0.11 μg/g, respectively) and then leveled off. In the case of caffeic acid greater yields were achieved when time increased from 2.5 to 10 min and then leveled off (Fig. 4D). Each bioactive compound may have different microwave absorbing properties or a different dielectric permittivity which may affect its selective heating explaining the differences in the extraction time [36]. Zhang et al. [14] noted that during MAE, extraction time significantly affected the recovery of six phenolic compounds from *A. blazei*. Future RSM studies are encouraged to optimize and investigate the interaction effects between different extraction parameters during MAE. The parameters that are suggested to be included in an RSM study are the particle size of the sample, microwave power, and extraction time. Even though the particle size of the sample had no effect on the recovery of caffeic and chlorogenic acid in the range that it was examined, smaller particle sizes (i.e., 0.50 mm, 1.00 mm) may positively affect the recovery of the phenolic acids. Phenolic acids are mainly found in bound forms (i.e., esterified and glycoside-bound) [36]. Therefore, it could be assumed that when smaller particle sizes will be used, the recovery of the phenolic acids might be enhanced since a larger surface area will be exposed to microwave power which may lead to the cleavage of the bound phenolics.

### 3.6 Comparison of different extraction methods

**Fig. 5:**
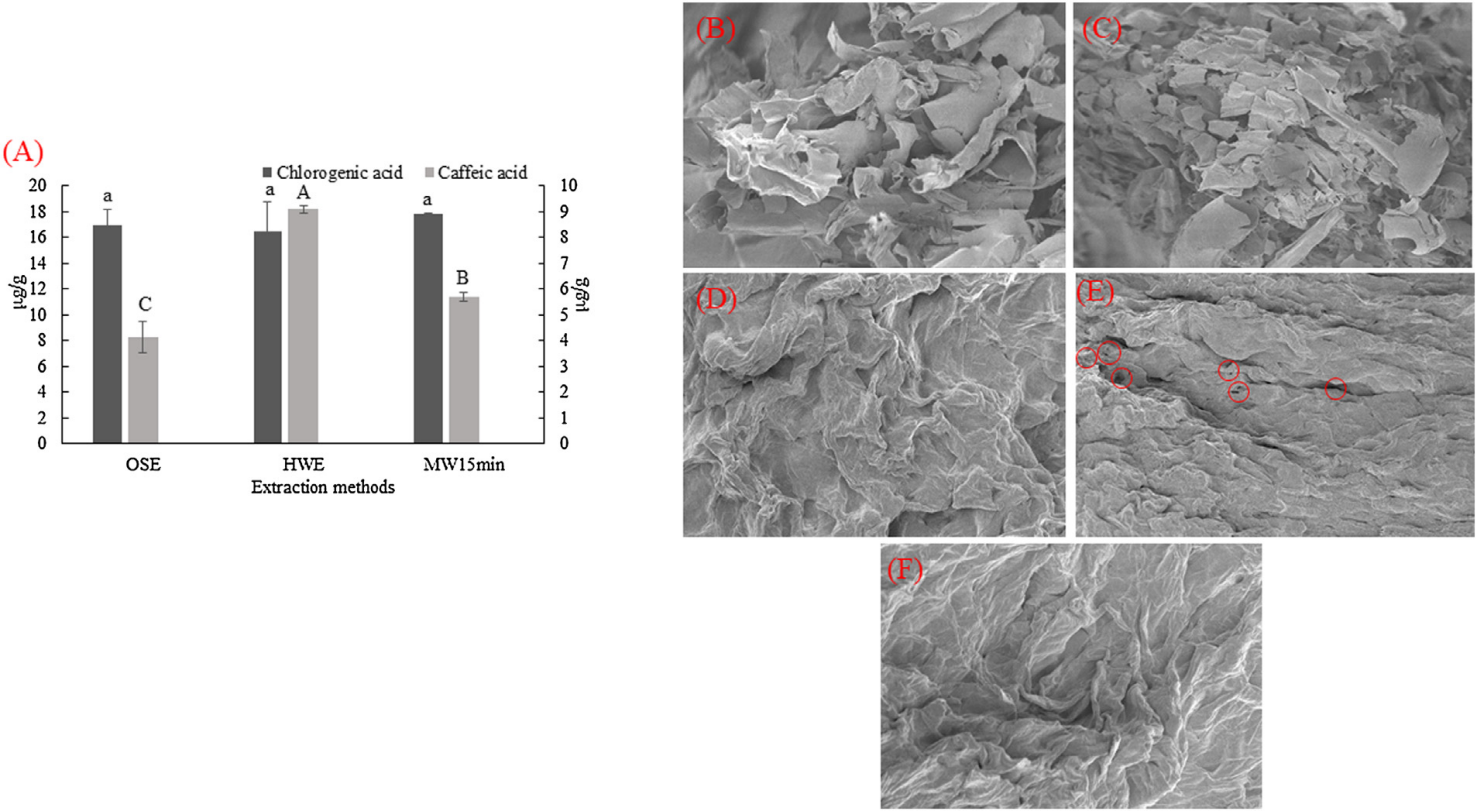
Effect of different extraction methods on chlorogenic acid (primary left vertical axis) and caffeic acid (secondary right vertical axis) (A); scanning electron microscopy images (SEM) of: freeze-dried mushrooms without extraction (control 1) (B), freeze-dried mushrooms without extraction placed in the oven at 60 °C (control 2) (C), mushroom residues after organic-solvent extraction (OSE) (D), mushroom residues after microwave-assisted extraction (MAE) (E), mushroom residues after hot-water extraction (HWE) (F).

The extracts obtained at the optimal MAE were compared with those obtained from a HWE (extraction temperature of 45 °C and extraction time of 5 h) [25] and an OSE (40 % (v/v) ethanol, extraction temperature of 90 °C, and extraction time of 1 h) [26]. The extraction method significantly affected the TPC, antioxidant capacity, and caffeic acid (Table 2, Fig. 5A), while there was no effect on the recovery of chlorogenic acid. The MAE resulted in extracts with similar TPC values (11.23 ± 1.05 mg GAE/g) to those obtained from the HWE (11.29 ± 0.12 mg GAE/g), and higher than those obtained from the OSE (3.68 ± 0.55 mg GAE/g). The HWE resulted in extracts with 23 and 26 % higher DPPH and CUPRAC values, respectively, compared to the MAE, while the OSE extracts had the lowest antioxidant capacity (Table 2). During the MAE, microwaves penetrate the sample at the speed of light, causing heat generation within the samples in short treatment times. The generation of heat results in pressure increase inside the cells, leading to cell wall disruption [23,37]. Cell wall disruption facilitates solvent entry which solubilizes the bioactive compounds, hence, the release of bioactive compounds into the extraction solvent. On the other hand, in conventional extraction techniques, heat is transferred by convection and conduction, thus longer extraction times are required. Similar results were reported by Zhang et al. [14] who reported that MAE resulted in extracts with higher gallic acid, protocatechuic acid, catechin, syringic acid, myricetin, and quercetin contents than HWE.

**Table 2:** Comparison of different extraction methods (organic-solvent extraction (OSE), hot-water extraction (HWE), and microwave-assisted extraction (MAE)). The results are expressed as mean ± standard deviation (n = 3).

Extraction method	TPC (mg GAE/g)	DPPH (mg TE/g)	CUPRAC (mg TE/g)
OSE	3.68 ± 0.55^b^*	4.16 ± 0.88^c^	8.04 ± 1.08^c^
HWT	11.29 ± 0.12^a^	8.87 ± 0.03^a^	19.51 ± 0.63^a^
MAE	11.23 ± 1.05^a^	7.19 ± 0.24^b^	15.43 ± 0.56^b^

* Values at the same column followed by different superscript letter within the same column are significantly different at *p* < 0.05 according to ANOVA and Duncan’s test.

SEM was employed for observing potential morphological changes on the surface of mushroom residues after the extraction (Fig. 5B–F). Before SEM analysis mushroom residues (samples after the extraction) were dried to constant weight at 60 °C. To exclude any effects of drying temperature on the morphology of the samples, two controls were used. As it is seen in Fig. 5B and C, drying temperature had no effect on the morphology of the freeze-dried mushrooms. The images obtained from the SEM showed that all the extraction methods let to the deformation of mushroom samples to a different extent (Fig. 5D–F). In the case of MAE, some cavities were observed (Fig. 5E) which might be due to microwaves that heat the inner part of the samples leading to pressure increase inside the cells which may result in cell wall disruption [23]. To sum up, MAE required significantly shorter extraction times than HWE and OSE for the preparation of extracts enriched in polyphenols and high antioxidant capacity.

## 4 Conclusions

The linear effects of different MAE parameters (particle size of the sample, solid-to-liquid ratio, microwave power, and extraction time) on TPC, DPPH, CUPRAC, chlorogenic acid and caffeic acid of shiitake mushroom extracts were investigated. At a higher microwave power (500 and 600 W) chlorogenic acid was decomposed to caffeic acid due to the temperature increase in the samples. This is the proof that chlorogenic acid is a thermally unstable compound. Future studies should be conducted employing RSM to investigate not only linear but also interaction and quadratic effects between different MAE parameters on the recovery of polyphenols (total and individual) and antioxidants from shiitake mushrooms. The parameters that are suggested to be included in the RSM design are particle size of the sample, microwave power, and extraction time. Given that the independent variables investigated in this study affected the five responses (TPC, DPPH, CUPRAC, caffeic acid, and chlorogenic acid) differently, the desirability option is recommended to be used in the future RSM studies. The desirability option has the advantage of providing the optimal values for several responses concomitantly. Among the three different methods examined, MAE proved to be an efficient extraction technique for the preparation of mushroom aqueous extracts enriched in polyphenols and antioxidants.

## CRediT authorship contribution statement

**Wen Xiaokang:** Investigation, Methodology, Writing - original draft. **James G. Lyng:** Funding acquisition. **Nigel P. Brunton:** Funding acquisition, Writing - review & editing. **Lydia Cody:** Visualization, Writing - review & editing. **Jean-Christophe Jacquier:** Writing - review & editing. **Sabine M. Harrison:** Writing - review & editing. **Konstantinos Papoutsis:** Conceptualization, Visualization, Methodology, Writing - original draft, Writing - review & editing, Supervision.

## Declaration of Competing Interest

The authors have no conflict of interest to declare.
